# Pharmacosex: Reimagining sex, drugs and enhancement

**DOI:** 10.1016/j.drugpo.2020.102943

**Published:** 2020-12

**Authors:** Leah Moyle, Alex Dymock, Alexandra Aldridge, Ben Mechen

**Affiliations:** a Royal Holloway, University of London, Egham Hill, Surrey, TW20 0EX, United Kingdom; b Goldsmiths, University of London, United Kingdom; c King's College London, United Kingdom

**Keywords:** Sex, Drugs, Enhancement, Pleasure

## Abstract

**Background:**

The use of drugs in sexual contexts is receiving closer attention in the media, public health bodies and communities than ever before. However, research to date is most often concerned with the sex-related drug use of lesbian, gay, bisexual, transgender and queer (LGBTQ) populations, and particularly men who have sex with men (MSM) engaging in ‘chemsex’. Against a backdrop dominated by public health and medical science perspectives, this article seeks to move beyond prevailing sex on drug discourses characterised by risk and harm, or pleasure. Drawing on an expansive notion of enhancement, we explore intersections between drug consumption and sex via the concept of ‘pharmacosex’: the ways in which wider populations experiment with a range of illicit drugs that modify and enhance their sex lives in the context of broader processes of the pharmaceuticalisation of sexuality.

**Methods:**

Drawing on two empirical studies comprising a virtual ethnography and 45 interviews with participants across a range of gender and sexual identities who regularly combine sex and drugs, this article contributes to the growing body of research that attends to the materiality of drug consumption practices in relation to the historical and social contexts from which they emerge.

**Results:**

Our participants reported variegated and complex modes of enhancement in relation to a wide range of psychoactive substances. Participants described enhanced emotional connectedness, bodily sensations, disinhibition and desire, but they also discussed how sex enhances drug experiences. As important but currently neglected in research literature were the therapeutic dimensions of drug-taking reported, which cannot be neatly distinguished from purely hedonic motivations. While enhancement was also experienced by participants in more challenging ways in relation to shame, regret, risk and/or harm, these experiences simultaneously afforded space for the emergence of innovative practices of risk-management, safety and care.

**Conclusion:**

This study exposes the diversity of practices and meanings sex-related drug use hold for participants, but also demonstrates the paucity of biomedical conceptions of sexual enhancement limited to stamina, function and libido, and the need for a more expansive approach. The study also raises questions about the extent to which contemporary discourses of self-improvement have come to ‘inhabit’ sexuality in the twenty-first century, and the role drugs might play in this context. By shifting the gaze from pathology to enhancement and exploring the plurality of practice, we can better understand the motivations for engaging in sex-related drug use, thereby circumventing knee-jerk counterproductive enforcement and policy responses.

And I guess I remember that everything was extra intensified…but like I knew, you know, during that whole phase, that sex and drugs was a great combination.

(Luna, F31, Heterosexual, London - Study 1)

## Background

### Chemsex and beyond: From pathology to pleasure

This article explores the intersection of drug use and sexual practice – referred to here as sex-related drug use – through the lens of enhancement. In recent years, sex-related drug use has received substantial popular (e.g. Bishop, 2018; Strudwick, 2020), policy (e.g. HM Government, 2017; Public Health England, 2015) and research attention (e.g. Hakim, 2019; Moncrieff, 2018). This attention largely centres on men who have sex with men (MSM) engaging in ‘chemsex’. The term ‘chemsex’ refers to a distinct set of sex and drug-related practices occurring among gay, bisexual and other MSM (Hakim, 2019; Race, 2009; Race, 2018). Chemsex involves the intentional use of substances – specifically methamphetamine, mephedrone and GHB/GBL - before or during sex, so as to facilitate, sustain and/or enhance the experience (Bourne et al., 2014). Instances of chemsex are often organised via geospatial ‘hook-up’ apps and are likely to involve sex with multiple partners (MENRUS, 2018).

Media depictions of chemsex – typically sensationalising and alarmist (Hakim, 2019) – position the phenomenon as an ‘epidemic of drug-fuelled gay sex’ (Dotheé, 2020; see also Hodgson, 2019). The combination of GHB/GBL, methamphetamine and mephedrone are described as ‘intensely addictive’: ‘[u]sers are consumed by soaring highs and then swallowed by the darkest lows’ (Dotheé, 2020). Echoes of media depictions are also evident in some of the academic literature on chemsex, which mostly comprises public health and medical science perspectives. Chemsex is positioned as a threat to public health (Hibbert, Brett, Porcellato, & Hope, 2019; Stevens, Moncrieff, & Gafos, 2020) requiring urgent professional intervention. Emphasis is placed on links between drug use and ‘risky’ sexual behaviour (Puffal et al., 2018; Hegazi et al., 2017) and chemsex engagement is (in part) ‘explained’ by a unique kind of cultural trauma experienced by gay and bisexual men (Stuart, 2016a; Stuart, 2016b).

Against this backdrop, some scholars (e.g. Hakim, 2019; Race, 2009; Race, 2018) have moved beyond explanatory frameworks that diagnose chemsex as an individual or group-level pathology. Instead chemsex is understood as a distinct set of cultural practices that ultimately emerge from particular historical, social and material contexts. By attending to the complex processes by which *pleasures* are generated in chemsex events, these scholars eschew pharmacologically determinist accounts of drug ‘effects’ (i.e. as resulting solely from the pharmacological properties of substances, rather than the events through which they are experienced) and contribute to a growing critical drugs literature that considers the materiality of drugs themselves to be ‘emergent and contingent’ (Pienaar, Murphy, Race, & Lea, 2020a, p. 2). For Race, (2008, p. 421), ‘[p]leasure is not the antithesis of self-regulation and safety, but the medium through which certain shared protocols of safety take shape’.

Despite the focus on chemsex, it is by no means the case that sex-related drug use is restricted to MSM having sex on methamphetamine, mephedrone and GHB/GBL. However, research beyond chemsex generally remains concerned with the sex-related drug use of lesbian, gay, bisexual, transgender and queer (LGBTQ) populations (e.g. Pienaar, Murphy, Race, & Lea, 2020a; Pienaar, Murphy, Race & Lea, 2020b; Ristuccia, LoSchiavo, Halkitis, & Kapadia, 2018; Parent et al., 2020). When studies have recruited participants across a broader range of social groups (e.g. Bellis et al., 2008; Lawn, Aldridge, Xia, & Winstock, 2019; Palamar et al., 2018; Rawson, Washton, Domier, & Reiber, 2002; Sumnall et al., 2007), they often employ a 'pathology paradigm' (Mugford, 1998) and explore drug use only ‘in the context of sexual risk-taking’ (Sumnall et al., 2007, p. 525) (see also Bellis et al., 2008; Rawson, Washton, Domier, & Reiber, 2002). Other studies focus on the ‘measurable’ effects of individual drugs on specific aspects of the sexual experience (Lawn, Aldridge, Xia, & Winstock, 2019; Palamar et al., 2018) – in doing so enforcing normative conceptions of sexual ‘function’ (e.g. ‘ability to achieve and maintain: an erection (if male)/moistness (if female)’ (Lawn, Aldridge, Xia, & Winstock, 2019, p. 724) – without attending to drug consumption contexts and their potential to augment experiences (Hakim, 2019). As a result, the processes by which drug ‘effects’ emerge as complex ‘intra-actions’ (Barad, 2007) among the range of ‘actors, contexts and practices’ (Pienaar, Murphy, Race, & Lea, 2020a, p. 2) immanent to each and every instance of sex-related drug use are obscured.

### From pleasure to enhancement

Although attending to pleasure has undoubtedly been productive in adding nuance to the binary narratives characterising sex-related drug use, to focus solely on this aspect of the drug use ‘event’ (Dilkes-Frayne, 2014) would also be reductive as it fails to account for the diverse and sometimes ambivalent ways in which individuals present their experiences of sex-related drug use. In this article, we draw on the notion of *enhancement* so as to attend more fully to the range of effects, intensities, and desires reported by our participants.

While medical sociology has highlighted the limitations of biomedical definitions of enhancement that point to interventions aimed at improving the ‘form and function’ (Conrad & Potter, 2004) of bodies ‘not in need of repair’ (Bostrom & Sandberg, 2009), this critique has often been neglected in sexualities studies. To date, characterisations of individual drugs that have the effect of erotic enhancement typically focus on more prescriptive functions and make clear delineations between drugs that augment physical function and capacity, and those which disrupt libido and desire. For example:

‘sexual enhancers are drugs that enhance (normal/abnormal) erectile function and aphrodisiacs, which increase sexual arousal and desire’

Van de Ven, Mulrooney, & McVeigh (2020, p. 3).

To suggest that the relationship between sexuality, drugs and enhancement can be reduced to a ‘measurable’ set of functions and use-value forecloses the possibilities substances might play in disrupting normative, biomedical conceptions of enhanced sex through their intra-actions with other ‘bodies, technologies and forces’ (Dennis, 2019, p. 21). There is also, perhaps, a false differentiation made in some literature (e.g. Tiefer, 2008) between recreational and therapeutic use of ‘sexual enhancers’, in which the hedonic qualities of sex-related drug use are considered distinct from experiences of self-medicating, where drugs are used for the reparation or resolution of sexual inhibitions or problems. The analysis presented here suggests that drug-related sexual practice, where enhanced sensation, desire and pleasure are drivers for participants’ use of drugs, requires a more expansive conception of enhancement in which these binaries are unpacked. We employ a ‘scavenger’ (Halberstam, 1998) approach to theory common in queer methodologies, where traditional disciplinary boundaries are refused, and ideas conventionally treated as being ‘at odds’ are brought together. Taking up insights from science and technology studies, queer theory, and the work of Paul B. Preciado enables us to think through some of the problems with mechanistic conceptualisations of sexual enhancement, and advance a more capacious approach to sex-related drug use.

Preciado's work of auto-theory, *Testo Junkie*, provides just such a counterpoint to biomedical characterisations of drugs as sexual enhancers, navigating the space between substance use as self-medication and as experimentation. The book charts the narrator's experiments with self-administration of testosterone as a project of ‘biohacking’: a reappropriation of the very biotechnologies that have produced the ‘technical subjectification’ (2008, p. 129) of sex and gender rather than as part of a medicinal ‘protocol to change sex’ (2008, p. 55). Preciado situates his experimentation with drugs within the context of the emergence of lucrative pharmaceutical markets for sexual enhancers in the twentieth century, in which sexuality becomes the subject of biopolitical management. In this purview, it is impossible to ignore that the concept of sexual enhancement itself relies on a series of historically contingent diagnostics prescribing what optimal sexual function, arousal and desire might look like. ‘Sub-optimal’ sexual function, arousal or desire has been subject frequently to pharmacological intervention, often dispersed along crudely gendered lines (Tiefer, 2008), and with prescriptive guidelines as to exactly what function is being restored. Race's work demonstrates that the panic over recreational use of ‘lifestyle drugs’ viewed commonly as sexual enhancers, such as Viagra in the early twenty-first century, was because it was perceived as a ‘gay’ drug used primarily by MSM to enhance sex rather than its prescriptive function of 'repair’ (2009, p. 5). However, the meanings attached to drug use as a form of sexual enhancement for women remains under-researched and perhaps more fraught still.

Crucially, Preciado's work also provides a previously neglected genealogical framing for the roles gender and sexuality have played in developing pharmaceutical somatechnical interventions (Mechen, 2020). Our conceptual cue for this article is taken from Preciado's work, adapting his term - ‘pharmacopornographic’ - to describe the ways in which pharmaceutical enhancements for sex have come to ‘inhabit’ (Preciado, 2008) sexual subjectivity in the twenty-first century. We argue that this process of inhabitation necessarily informs individual drug users’ motivations for using substances for enhancement, also providing a basis upon which illicit drugs are seen to reappropriate or imitate the effect of legal pharmaceuticals. We call the entanglement of processes of pharmaceuticalisation and reappropriation, and what recreational enhancement might mean in the context of these processes, pharmacosex.

Situating the ‘pharmacopornographic era’ as a distinctly late modern epoch in the history of sexuality, we argue that sexual enhancement through drug use must also be contextualised within broader narratives of self-realisation, personal growth and happiness, and technical and personal improvement (Giddens, 1992) that ‘encode an optimisation of one's corporeality to embrace a kind of overall wellbeing’ (Rose, 2001, p. 17). Narratives of neoliberal aspiration inevitably inflect participants’ reflections on what is gained from using drugs in a sexual context, and what sexual enhancement means to them. Discourses of sexual self-improvement are recognised as particularly forceful in governing women's choices (Gill, 2007; McRobbie, 2007), especially their consumption practices. While we do not eclipse questions of agency and resistance, our findings suggest that enhancing sex through drug consumption represents one of a multiplicity of possible consumer choices made by ‘sensation gatherers’ (Bauman, 2003) in the sexual marketplace of the twenty-first century.

## Methodology

This article draws on qualitative data from two studies conducted between 2018-19 in the UK. Importantly, both studies did not focus exclusively on the sex-related drug use of LGBTQ groups. While sexualities researchers often argue that this approach risks ‘lumping together’ (Kneale et al., 2019) diverse experiences and differentiated processes of marginalisation, both studies were designed to avoid the overattentiveness to LGBTQ drug use that has characterised almost all research on the subject to date, leading to stigma around drug use in general, and subsequent over-policing and marginalisation of these populations.

The principal study (Study 1 – AD, LM and BM) comprised an archival scoping exercise exploring the broader historical context of sex-related drug use. This was complimented by an empirical component reported on here, comprising a virtual ethnography (Barratt & Maddox, 2016) and semi-structured interviews with 31 individuals who had experience of sex-related drug use. Virtual ethnography consisted of ‘digitally tracing’ interactions among people who use drugs on online forums such as *Bluelight* and *Reddit*, and locating descriptions of sex-on-drug experiences via these mediums and in the ‘psychoactive vaults’ on the website *Erowid*. Virtual ethnography has been noted as particularly fruitful by researchers interested in drug use as providing new horizons for field observation (Decary-Hetu & Aldridge, 2015). As we note in the findings below, almost all participants interviewed in Study 1 reported using these online mediums to research the effects particular drugs might have on sexual enhancement. Interviews focused on exploring the types of drugs selected and the ways these were utilised to modify and enhance sexual encounters. It was left to participants to decide what they considered to be a ‘drug’, and, for the most part, they chose to focus on experiences involving illicit substances (e.g. cocaine, cannabis, MDMA).

Study 2 (AA) comprised 14 in-depth interviews, again with participants who had experience of sex-related drug use. Similar to Study 1, participants decided what counted as a drug and almost exclusively focused on experiences involving illicit substances. At the outset of each interview, participants were asked to describe an (ideally recent) sex-on-drug experience. They were then asked a series of follow-up questions to elicit details about the contexts, meanings and effects associated with that experience. Participants were then asked to describe other sex-on-drug experiences so as to build an extended account of sex-related drug use over their lifetimes. Demographic and drug use history data were collected at the end of each interview.

In Study 1, the majority of interviewees were recruited via a call for participants advertised on *Twitter* and drug discussion forum *Bluelight*. Several participants contacted researchers directly to enquire about involvement after an interview Dymock undertook about the research on the BBC Radio 4 show, *Women's Hour*. All participants in Study 1 were offered £10 as compensation for their time. Study 2 participants were recruited through a combination of purposive and snowball sampling – techniques typical for accessing ‘hidden populations’ like people who use drugs (e.g. Barratt, Ferris, & Lenton, 2015; Boys, Marsden, & Strang, 2001). Participants in Study 2 were not offered financial compensation. Both studies received ethics approval from their respective institutions (Royal Holloway, University of London and University of Cambridge) and adhered to the standard code of practice common to virtually all social research: use of information sheets and informed consent forms, informed voluntary participation, treating disclosures as confidential and using these anonymously, and secure data handling and storage. In line with ethical guidelines published by *Bluelight*, researchers in Study 1 obtained permission to advertise and use data from this forum. Given that data from the virtual ethnography included is freely available from the surface web, we have included the title of forum posts, but omitted the author's username and any other identifying features.

All interviews were audio-recorded and transcribed verbatim. Transcripts were analysed thematically (Braun & Clarke, 2012) using qualitative data software NVivo. In Study 2, thematic analysis was complimented by narrative analysis techniques (Riessman, 2005). Thematic analysis involved identifying patterns across datasets, and the narrative element explored how interviewees arranged these themes to form an extended account of their experiences of sex-related drug use over time.

The data and analysis presented below seeks to complicate current narratives around sex-related drug use through exploring participants’ talk around the variegated and complex set of enhancements that emerge in relation to pharmacosex. We move beyond normative/biomedical approaches to enhancement in favour of a more exploratory and expansive conceptualisation. To achieve this, we draw on the notion of enhancement as it pertains to the themes of ‘chemical connection’ and communication, physical sensations and sexual openness, disinhibition and sexual experimentation, and ‘therapeutic’ drug use. To conclude, we consider how our analysis might contribute to burgeoning research on sex, drugs and enhancement, as well as literature on chemsex and harm reduction.

## Findings

### Demographic diversity

Across both studies, interview participants were aged between 21-65 years. 43 were based in multiple cities across the UK and two were based abroad. In a departure from the majority of existing literature which focuses disproportionately on cis gay men, our research achieved an even gender split. 22 of our participants were cis-male, 21 cis-female, and two identified as non-binary. Our sample was, however, much less representative when it came to self-defined ethnicity, with only five participants identifying as ‘mixed-race’ and the vast majority (n=40) describing themselves as white. Of these, the larger number described themselves as British (n=30), and a smaller representation as Jewish (n=3). The remainder were European and American. Participants identified across a range of sexual orientations, with some selecting more than one category. Highlighting the appeal of sex-related drug use beyond LGBTQ communities, the most represented sexual orientation was heterosexual (n=18), followed by bisexual (n=14), heteroflexible (n=6) and pansexual/panromantic (n=6). A smaller number described themselves as gay (n=3), queer (n=2), bisexually straight (n=1), demisexual (n=1) and asexual (n=1).

### Range of drugs and types of use

MDMA was by far the most commonly used drug with sex across both studies. 91% (n=41) of our total interview participants (n=45) reported having used this substance in sexual encounters. Cocaine had been used by 51% of our sample (n=23), followed by cannabis (64%, n=29), ketamine (36%, n=16), LSD (32%, n=10), GHB/GBL (27%, n=12) amphetamines (20%, n=9), and mephedrone (19%, n=6). Less commonly used drugs included 2C-B (n=5), methiopropamine (MPA) (n=3) and nitrous oxide (n=2). Two participants reported using Valium in sexual contexts. The same substances were broadly found as most commonly cited as enhancing sex in the virtual ethnography, although other substances, which interview participants sometimes lamented were difficult to access, included novel psychoactive substances such as 5-MeO-DIPT (‘Foxy Methoxy’).

Analysis of the ways in which drugs were used for sex in Study 1 indicated four dominant types of user. Most common were *recreational drug users* – those who had combined sex and drugs unintentionally (e.g. an MDMA user who has sex while high, and then goes on to utilise this substance again once they had ‘learned’ (Becker, 1953) its positive enhancing effects). The second largest group were individuals who engaged in sex-related drug use in *BDSM and sex party contexts -* both in private settings and the night time economy. This was followed by *experimenters –* experienced drug users who were more likely to experiment with research ‘chems’ and dose in order to alter experience and effect. The final category was *chemsex*. Despite the research and policy focus on this particular form of sexualised drug use, this was the least prevalent form of use across our studies. The users described are offered as ‘ideal types’ (see Segady, 2014) rather than rigid categories, with our respondents commonly drifting across or engaging in more than one category of use.

### ‘Chemical connection’ and communication

The perception that certain drugs had the capacity to enhance emotional connectedness in sexual contexts was common amongst participants. This finding is perhaps unsurprising given the prevalent use of empathogens in our sample, but the capacity for communication to enhance sex also demonstrates the paucity of biomedical conceptions restricted to physical performance, and the false distinction between therapeutic and recreational use. MDMA has been described as psychiatry's antibiotic (Sessa, 2005; Sessa, 2007) used as a tool to assist with therapeutic interventions due to its variety of pro-social effects. This characterisation might itself be described as a reappropriation of licit ‘medicinal protocol’, outlined by Preciado. In a recent study of couples’ use of MDMA, Anderson et al. (Anderson, Reavey, & Boden, 2019) explore how these effects enable ‘boundary work’ (p.10), enhancing existing feelings of closeness in romantic relationships. This finding was reflected in the experiences of participants in the current studies who had taken MDMA/Ecstasy with their partners. In the context of pharmacosex, MDMA appeared to be doing the work of facilitation that we might expect to be undertaken by a couples’ therapist, providing a symbolic mediator for exploratory communication about sex that participants tended to view as crucial to improving it:

I think the other thing is that you're able to talk much more freely about sex, and I think everyone has a reticence to talk about things they like or really specific fantasies that you're always worried about, “Oh, is this weird?” and I find when you're high you're really able to talk about that and you're able to communicate it better and set those scenarios up better...I think both of us have, at the peak of MDMA, spoken about sexual things we would like to try…

(Abel, M30, Heterosexual, London – Study 1)

When we do MDMA, we tend to have like a discussion topic of the evening, if we're at home...and quite often it's something that we wouldn't think to talk about if we weren't high.... This time it happened to be that strap-ons were the topic of conversation (laughs). So, we chatted about it for ages, I can't remember much of what we said because we were really high. But basically, we came to the conclusion that it was something we wanted to do more of together.

(Pink, F24, Bisexual, Cambridge - Study 2)

Interview data suggests that the value of empathogens went beyond these more familiar themes of connectedness and communication. Our participants also emphasised the capacity of these drugs to enhance their sex lives through easing the anxieties and awkwardness commonly linked to sex with a new partner. This finding reflects some of the observations made by Alexander Shulgin when he resynthesized MDMA. He noted that the compound made a ‘close’ and ‘very intimate interaction’ possible (MAPS, 2002), thus promoting what he labelled an ‘untenseness’ during sex with new partners as well as existing ones. In the current studies, Chris (NB22, Pansexual/Demisexual Cambridge - Study 2) recalled a ‘one-night stand’ involving MDMA that challenged their belief that their capacity for sexual attraction depended on the presence of a prior emotional connection. They believed MDMA enhanced their ‘*conversation, confidence, and willingness to listen*’, leading to a deep ‘*chemical connection*’ with someone they had met hours earlier.

The use of drugs to enhance connection and ease awkwardness during sex was not limited to MDMA/Ecstasy. Another participant described using cannabis with the intention of facilitating a chemical connection with a sexual partner she deemed herself otherwise incompatible with:

[W]e really had nothing in common...but being high was something we both liked to do. And it made hanging with him easy, it made the sex better... or at least easier, and less awkward [laughs].

(Libby, F25, Bisexual, Cambridge – Study 2)

Elsewhere, Hanna recalled a first-time encounter involving 2C-B, a psychedelic substance with properties similar to MDMA (Dean, Stellpflug, Burnett, & Engebretsen, 2013). For Hanna, this encounter generated some of the best sex they had ever had:

And the sex was like… it was, amazing. It was so good. And I remember looking at him, and him looking at me, and like… us just fucking, and I was like, what is this? This feels so good. It was, like, so intense, and he was like, I know, something's different. …And I just woke up the next day like what the fuck?…I was like, I feel like I've known you? It's like getting to know the soul before you know all the other stuff that makes a person a person. It's like you get to know the innermost personality before the extra things.

(Hanna, NB23, Pansexual/Panromantic, Cambridge – Study 2)

The participants quoted above appeared to value the chemical connections that emerged in relation to their use of MDMA, cannabis and 2C-B, and the capacity of a shared drug experience to elevate sexual encounters beyond a physical meeting of bodies. However, others experienced chemical connections in more challenging ways. Rose, for example, expressed ambivalence over the enhanced familiarity and trust that MDMA appeared to promote:

I've done things with you that I've never done with anybody else, I felt very exposed and vulnerable in some ways but at the same time probably with the MDMA it gave me this level of trust and comfort that I could explore these things with you.

(Rose, F24, Heteroflexible, Liverpool - Study 1)

Others articulated concerns over the perceived artificial nature of chemical connections (Aldridge, 2020). Laura, for example, recalled a first-time sexual encounter involving MDMA in which she experienced a strong connection with the person she was having sex with. On their second meeting, however, Laura was unhappy to discover that this connection was no longer present and that she didn't ‘*really know or like*’ them very much (Laura, F25, Bisexual, Cambridge – Study 2).

The experiences described above can be contrasted with those where substances were perceived to stymy intimacy and connection, but enhanced sex in other ways. Cocaine, for example, was described by many as a drug that encouraged a more individualistic encounter due to what one participant termed its ‘ego inflation effect’:

Yeah, I would say coke makes me kind of, I guess, selfish. Like, quite pursuant of my own pleasure and less concerned about the other person and with the right person, if you're both in that mood, that can be quite, that's quite fun because you're just like very rawly fucking, that's good.

(Ziah, M42, Heteroflexible, London - Study 1).

Here, Ziah suggests that an inward focus during sex is not always an unwanted effect, and can in fact, if considered part of the drug ‘event’, enhance the experience with the ‘right’ sexual partner in a similar state of mind. Other participants - again notably women - reported very different experiences with cocaine to that relayed above. One associated it with *‘cold mechanical’* sexual encounters (Lily, F25, Heteroflexible, London - Study 1), while another felt it resulted in ‘*sleazy porn sex*’ (Suzi, F25, Bisexual, Glasgow - Study 1). Some participants – mostly cis-women – appeared comfortable articulating the value of drug-enhanced sex only insofar as they felt it might seem ‘justifiable’ because it facilitated intimacy and connection beyond the drug event, whereas participants who identified as men were far more likely to describe their experiences in purely hedonic terms.

### Physical sensations and sexual excitement

Previous research focusing on MSM populations has identified the desire to enhance or transform the bodily and affective experience of sex through drug use (Pienaar, Murphy, Race, & Lea, 2020a). Our findings revealed that these enhancements and modifications were also sought after and valued by other social groups - and often the key driver for experimenting with substances in sexual contexts. One participant summed up the perceived benefits of MDMA as being ‘*a bit like turning the volume up... everything is like you just totally whack the volume up*’ (Theo, M56, Heterosexual, Brighton - Study 1). For those active in the BDSM and Kink scene, substances like GHB/GBL, nitrous oxide and MDMA/Ecstasy were suggested to have value in aiding what one participant described as a ‘*surrendering headspace*’ (Clio, F30, Bisexual, London - Study 1), which enabled physical relaxation and made practices such as anal sex not only possible but pleasurable. Drugs were also afforded the capacity to transform pain into pleasure. Robert described how MDMA enhances his BDSM play and alters the nature of pain:

I don't know, it changes the pain receptors and substitutes a lot of pain receptors for pleasure receptors…The best way I can describe it is that all the things that normally really hurt unbearably hurt really nicely…The huge rush or sensation you get from being hit hard instead of being in a screaming amount of pain, it is painful but that pain is sort of mixed up with a massive new release of dopamine or whatever...

(Robert, M65, Heterosexual, London - Study 1)

For MDMA/Ecstasy users outside this scene, our data again moves beyond established discourses of connection, empathy and intimacy (Anderson, Reavey, & Boden, 2019), with embodied pleasure and sensation clearly indicated by our sample. Sex on MDMA was described as ‘*sex magnified by one thousand’*, experienced by one user as ‘*waves and waves of pleasure*’ (Erowid Report 9233). Heightened sensation and tactility were frequently related as a benefit of combining sex and MDMA-like substances including 2C-B and Ecstasy.

Accompanying these benefits, participants with penises also described difficulties in achieving and maintaining erections with MDMA/Ecstasy, an issue also associated with mephedrone and cocaine and supported by existing research (Peugh & Belenko, 2001; Zemishlany, Aizenberg, & Weizman, 2001). Lack of erection did not, however, necessarily diminish participants’ overall experiences, as Zemishlany and colleagues conclude. Rather, these encounters instead tended to become more exploratory and less orgasm-driven. Participants often initially described sexual enhancement facilitated by drugs in the biomedical, ‘measurable’ terms explored earlier, an example of the ways in which Preciado suggests the biomedical diagnostic of sexual function has come to produce a ‘technical subjectification’ of sex that might be pharmacologically alleviated. However, for many participants this expectation was then overcome by discovering other, sometimes unexpected forms of pharmacosexual enhancement were facilitated. Instead of penis stimulation and sexual encounters being focused on (male) orgasm as an end-point, participants found pleasure elsewhere:

I associated [MDMA] for a long time with erectile dysfunction. And when I started dabbling with BDSM, it [MDMA] came into its own then. Because a lot of that is associated with the sensual side of sex, and taking your time really.

(Ian, M52, Heterosexual, Manchester - Study 2)

Orgasm is very different, difficult when you're on MDMA as well so it makes the sex very different in itself. It's less focussed on an orgasm or someone's orgasm or anything so it's kind of that's really enjoyable, to just enjoy someone's body without it being centred around it ending at a certain point.

(Arlo, M22, Heterosexual, Oxford - Study 1)

The data above demonstrate the range of modes through which pharmacosexual enhancement was experienced by participants, in spite of normative perceptions of erectile dysfunction as diminishing sex. These narratives also corroborate Race et al.’s (2016) finding that perceptions of enhanced physical sensations are not necessarily limited to the parts of the body ordinarily connected with sex.

### Disinhibition and sexual openness

Disinhibition is frequently invoked to understand the relationship between sex and drugs (Race, 2015). Chemsex research (e.g. Weatherburn et al., 2017) in particular has commonly explored the disinhibitive effects related to the combined use of mephedrone, methamphetamine and GHB/GBL, most often emphasising the risks rather than pleasures related to heightened sexual arousal (Kapitány-Fövény et al., 2015, p.276). Some of the experiences framed as risks in this literature, including pushing boundaries, rough sex, and hypersexuality (Ma and Perera, 2016) were in some cases alternatively presented as enhancements by our participants. GHB/GBL had the potential to ‘*push things along*’ getting you *‘into the sex*’ (Bruce, M60, Heteroflexible, London - Study 1) and facilitated a sense of feeling where ‘*everything has to happen right now*’ (Elsa, F26, Heteroflexible, London - Study 1). This embodied sexual excitement and sense of sexual urgency is unpacked below by Sonny:

The way that I describe it when I'm talking to people who haven't taken it is that it reduces inhibitions and it does it pretty evenly across the board. So, if I'm eating popcorn, it's the most delicious popcorn. If I'm having an argument, it's the most important argument I've got to have. And if it's sex, you know, it's urgent, you know it will happen, and it will happen with the consent of both people.

(Sonny, M40, Pansexual/Bisexual, London – Study 1)

Though GHB/GBL in particular is more readily associated with disinhibiting effects and their associated harms than other drugs (see ACMD 2007; Cavanagh and Smith, 2018; Kapitány-Fövény et al., 2015), significantly, our data suggests sexual disinhibition is associated with a much broader range of substances and beyond MSM populations, across the sexuality spectrum. For participants in the current studies, a variety of drugs (e.g. mephedrone, methiopropamine, ketamine, MDMA, cannabis) were linked to heightened sexual desire and loss of inhibitions around sex, which in turn enhanced willingness to engage in sexual experimentation (Sumnall et al., 2007). Emma, a 36-year-old heterosexual woman (London, Study 1), reported using mephedrone as an enhancement drug, which she described as producing a ‘*huge appetite for sex*’. Reflecting on his experience of using methiopropamine (MPA) and mephedrone, Cyril describes how they similarly worked to enhance sexual stimulation and libido:

So, I used to take mephedrone quite a lot. You know, go out to clubs and parties and stuff, take mephedrone and be in this very heightened state of kind of sexual desire almost, but not actually acting on it and doing it. And again...I enjoyed being this…just feeling almost like the sexual power throbbing through the veins in my body and, you know, it being there, this potential…

(Cyril, M41, Bisexual/Pansexual, London – Study 1)

Despite MDMA's reputation as a being culturally accepted as a ‘love drug’ rather than typically associated with sexual enhancement (Beck & Rosenbaum, 1994), interestingly, there was meaningful evidence of our participants experiencing increased sexual desire or ‘horniness’ whilst combining sex and MDMA. One participant explained that sex on MDMA, in her words, made her ‘obsessed’ with sex:

I can't actually take MDMA because I get too horny on it. I don't touch it.  That's why I don't take it because I get so sexual that unless i've pre-planned to have sex with somebody I don't want to take it because otherwise I feel like I'm a sex pest.

(Clio, F30, Bisexual, London, Study 1)

Clio was not alone in making this claim, which was replicated by several other respondents. Another participant, Max (M37, Bisexual, London - Study 1), for example, described ‘*walking around [while on MDMA] just thinking that everybody…is sexually attractive’*, while Robert (M65, Heterosexual, London - Study 1) claimed MDMA turned them into ‘*a complete masochist slut*’.

Accompanying intensified sexual desire, another common theme related to the potential for drugs to enact increased experimentation as creativity and confidence grew. Drugs were seen to reduce feelings of self-consciousness enabling participants to be more sexually ‘open’:

I mean that is probably my main motivation, my driving factor behind me having…doing sex whilst on drugs. It's the…I really enjoy the complete loss of inhibition, deliberately putting myself into this state where I know that I'm going to be incredibly sexually open and, dare I say it, like libertine and debauched? And it's almost like taking those drugs to enable that state.

(Cyril, M41, Bisexual/Pansexual, London – Study 1)

Just because, like my journey with drugs has always been about letting go and I think the reason why it... Like, I have a very strong sense of control of myself and I thought that that would be good, with sex, to be able to let go. And with most drugs, I feel that I've left me behind, in like, you know, the control part of me.

(Nadiya, F48, Heterosexual, Iraq - Study 1)

Other participants spoke of ways in which drugs could assist them to push boundaries, allowing them to experiment with practices normatively considered niche or taboo such as erotic asphyxiation using nitrous oxide, and catheter insertion, thereby pushing at the limits of existing repertoires:

Maybe you're pushing your boundaries a little bit further, and that's kind of fun, it's fun to be...like put yourself maybe, not at risk in a dangerous sense but, you know, "Oh, am I comfortable with this? Well I am now, let's see how far we can go".

(Lily, F25, Heterosexual, London - Study 1)

As Lily suggests, the potential for substances to support sexual experimentation therefore bolstered respondents’ ability to act on more adventurous sexual desires. Sexual excitement and feelings of ‘horniness’ experienced therefore had a broader appeal.

It may be tempting to dismiss narratives of sexual experimentation as further evidence of neoliberal techniques of self-improvement. However, we suggest the experiences of participants frequently resist this determinist characterisation. Instead, our data suggests that pharmacosex often registers as a counter-movement against what were often perceived as self-imposed sexual constraints.

### Therapeutic use

While the relationship between sex, drugs and enhancement is most commonly understood as constituted through performance, libido and sensation, participants’ talk often slipped between discussing substance use for reparative means, and for pleasurable experimentation. For many participants, discussion of drug use with therapeutic dimensions were ultimately linked to enhanced sexual experiences, and to break down this false binary requires a more capacious understanding of enhancement. This finding is usefully contextualised within the long history of psychedelic therapy (Dyck, 2008) and in the current ‘psychedelic renaissance’ (Sessa, 2012; Bøhling, 2017), in which in controlled trial settings, the use of psilocybin, ketamine and MDMA have proven remarkably successful in treating anxiety, depression, Post Traumatic Stress Disorder (PTSD) and other mental health conditions as an adjunct to guided talking therapy sessions (Nutt, 2019; Torjesen, 2014). While there is very little research to date specifically concerned with how psychedelic therapy has been or might be used to treat psychosexual problems (Dymock, Mechen, & Moyle, 2019), given the volume of media coverage recent clinical trials at Imperial, Oxford and King's College London have received in the UK (e.g. Jacobs, 2019; Southworth, 2020), it is perhaps unsurprising that participants appeared cognisant of the therapeutic effects of individual substances, and often self-administered to achieve these ends.

Some participants explicitly discussed the therapeutic benefits of substances for sexual enhancement:

I do suffer from depression and things like that and so it's something I find really helpful for my mental health to do every now and again with LSD. I think that doing that with a partner can be nice as well…so I'm very comfortable with that and see that as a really therapeutic, I see it as a therapeutic thing.

(Jen, F32, Bisexual, London – Study 1)

Psilocybin…the second time it happened is that, from that first experience, we learnt that, with another partner, it can be a great tool to learn sexual...the social pleasure of others, it's a communication tool, and in that way the second time we used psilocybin it was functional, it was like, "Oh this will be good for our sex life".

(Arlo, M22, Heterosexual, Oxford – Study 1)

For one participant, using amphetamines mitigated the effects of ADHD and enabled her enjoyment of sex:

I think people have this conception that it [Speed] literally speeds you up in terms of you feel like you're racing, but actually weirdly when you take it for ADHD it actually just feels like you slow down. You feel a sense of calm, and the stuff that would normally make you really anxious dissipates. So, all of that when you put it in a sexual situation just makes it an easier thing.

(Elsa, F26, Heteroflexible, London - Study 1)

In other instances, participants’ talk less explicitly cited the therapeutic benefits of sex-related drug use, but nonetheless borrowed from therapeutic culture (Rose, 1990) to discuss processes of enablement:

I had very negative sexual experiences as a child and have all sorts of blocks about my sexuality so I thought that maybe there'd be a drug out there that would, like, unlock those doors.

(Nadiya, F48, Heterosexual, Iraq - Study 1)

I am a survivor of sexual abuse and my primary interest in psychedelics and empathogens is their healing properties, particularly the ability to have sexual experiences while remaining present and fully embodied.

(Erowid Experiences Vault Report ID: 101526 *Quick Ascension of Sensual Mountain*)

More common amongst women, reference was made to previous difficult sexual experiences that they had sought to overcome through the disinhibitive effects of sex-related drug use. For some respondents, drugs did not only make sex possible, but enabled embodied, pleasurable, disinhibited sexual practice despite the sometimes-prohibiting nature of mental distress, psychological trauma and anxiety. In other instances, participants drew on processes of self-actualisation, where a more attuned sense of sexual selfhood, agency or embodiment had been uncovered:

I have such horrendous body issues. Like, I'm so…like constantly feeling disgusted with how fat I am or whatever, that weed is one of the only things that can take me out of that at the moment...I remember like having weed [and having sex], and thinking oh my god, I'm not thinking about my body, and I can just be in it, and I can just be present.

(Libby, F25, Bisexual, Cambridge – Study 2)

I'm generally speaking about GHB, although MDMA might do this as well... it helps me bring myself more into my body and enjoy pleasure more and not thinking so much about, "Am I doing this thing correct?"

(Alba, F22, Berkeley, Bisexual/Pansexual, USA - Study 1)

These less explicitly therapeutic narratives, nonetheless drawing on ideas of self-improvement and self-realisation, demonstrate that a more expansive understanding of the relationship between drugs and sexual enhancement must take into account the underpinning biopolitical logics of pharmacosex. The use of drugs in many cases conformed to the historical diagnostics of women's difficulties in achieving what they perceived to be ‘optimal’ sexual arousal or desire, rather than a focus on physical ‘function’. Moreover, women interviewed were often more reluctant to frame their experiences purely through the lens of pleasure, and tended to ‘justify’ their drug use as pivotal to achieving sexual ‘self-optimization’ (Barker, Gill, & Harvey, 2018).

### Difficult experiences

As well as describing enhancements to their sex lives, our participants discussed difficult or challenging experiences of sex-related drug use. A notable number of participants reported feelings of regret and shame relating to the sexual encounters they had engaged in (Fisher, Worth, Garcia, & Meredith, 2012). While these experiences were not necessarily understood to be breaches of sexual consent, participants nonetheless questioned whether they had actually enjoyed what had occurred. Particularly prevalent amongst participants who identified as women was concern about how their behaviour might affect how they were perceived by others:

Well as I say…as I sort of mentioned before, the biggest one is…you know sometimes you get shameful feelings afterwards because you look back and you're like, “Oh my God everyone must think I'm so annoying or a terrible slut,” or, “Oh my God what have I done? I've just…did I even enjoy that?” "Why did I play with these people?”...So I would say that's a definite negative.

(Isla, F37, Bisexual, London, Study 1)

As we have seen, for some, drugs provided a ‘chemical crutch’ for pushing personal boundaries and engaging in behaviours that they may not have otherwise considered. By example, Isla describes various sexual encounters involving GHB in which she participated in more ‘extreme’ sex acts that she later expressed reservations about:

I'd probably get in a BDSM context I've probably been hit harder when I've been higher because, you know, maybe I'm like, “Do it harder,” because you want more sensation. Which at the time hasn't been a problem but then you know you wake up and you've got these massive bruises and you're like, “Fucking hell, what's going on? What did I agree to?” Yes. And yes, occasionally I agree to things like I might…like once I agreed to someone putting a catheter in me and that was horrible. Like, that was awful, I wouldn't do that again.

(Isla, F37, Bisexual, London, Study 1)

For other participants, feelings of regret and shame extended to their choice of sexual partner:

It's like I went on a Tinder date with a guy and I'd taken like...I don't know, like maybe a couple of grams of Valium the night before after taking coke I guess, I can't remember, but I went on this date...and then ended up just being like “Oh, do you want to have sex?” and then we did and I was like “Oh I didn't really want to do that”, probably wouldn't have done that if I hadn't been on a lot of Valium, but yeah, Valium seems to be the drug that makes me go like "Yes", when actually I'm quite prudish and don't normally do that sort of thing.

(Lily, F25, Heterosexual, London – Study 1)

Existing research (e.g. Jozkowski & Wiersma, 2015) has tended to prioritise focus on legalistic concepts of consent to sexual activity, focusing on individuals’ drug use (most often alcohol use) as impairing the freedom and capacity to choose to engage in sexual activity. But as Lily and Isla's narratives indicate, there exists a lesser researched fine line between drugs enabling participants to push boundaries and experience enhanced, pleasurable sex, and situations in which sex-related drug use could make possible acts that are desired at the time, but are later regretted or considered shameful. It is important to note that the critical self-reflection described by participants was almost exclusively a gendered experience. Drugs could enable a temporary release from the self-consciousness more likely to affect women, but ultimately, these feelings could be persistent enough to return in the form of regret and shame. Participants who identified as women revealed anxiety about how their reputations amongst peers might be affected, and often reflected retrospectively on whether drug-enhanced sex had led them to push their limits beyond what they felt comfortable with sober.

### Harm reduction

Participants’ talk revealed comprehension of the risks associated with different drugs. Though many discussed drug use with friends, this often didn't extend to sex-related drug use. Participants also expressed a desire for harm reduction information that went beyond chemsex public health campaigns, which they felt were targeted exclusively at MSM and were usually limited to reducing the risks of taking the specific drugs linked to chemsex. Some participants also reflected on the history of public health campaigns around sex and drugs, and felt that the recent media scrutiny of chemsex skewed the reality of who engages in sex-related drug use, and their reasons for engaging in it:

Why is it always like gay men, their sexuality and practices are held up? And why is not we all, as human beings, get excited by dangerous activities and can encourage one another to get involved in dangerous activities. And it's just, you know, excitement is excitement...The media are just always going to pick up on gay men doing sex, always.

(Nadiya, F48, Heterosexual, Iraq - Study 1)

Many participants researched drugs and their potential effects on sex thoroughly online before experimenting, ensuring the set and setting (Zinberg, 1984) - that is, the role of individual differences and context - were as complimentary to the expected effects of the drugs as possible. Others were particularly cautious about dosage:

Yeah, like what kind of dosage should you take, what can you expect to happen, what are good things to do if it starts to go wrong, like what are good spaces to do this drug in, all that kind of stuff...But apart from that I never really remember seeing much information about sex, like everything I'd want in a discussion of like having sex on the drugs in the drug forums, but also my questions would normally be like, h"hh hh ow much acid should I take if it's my first time?"

(Lily, F25, Heterosexual, London - Study 1)

Given current media coverage of the risks and harms associated with GHB/GBL and sex (e.g. Dispatches 2019), it is notable that participants who reported using this substance were not only cognisant of the risks, but often reflected on the range of strategies used for risk-management and peer-care (Race, 2008) such as using stopwatches, minders and even controlling access to drugs:

At the parties I've been going to more recently, in the last few years, there's a sort of system where after you take it you write like the time and the dose on your arm...I think this started with one of my friends who's quite anal about the dosage because they'd seen too many people do too much and so he'd be in control of it. So, if you asked him for some more he'd check.

(Isla, F37, Bisexual, London - Study 1)

I wrote a couple of guides to various things which I've shared privately with friends…I wrote a guide to G harm reduction. I'm very, very cautious about sharing G with people so I kind of said, I send them the harm reduction stuff and then I generally supervise them if they're going with my supply.

(Stuart, M35, Heteroflexible, London - Study 1)

Several participants noted that their experience of the effects of particular drugs was purposefully augmented by engaging in sex. This was often in the context of ‘bad trips’ or to mitigate unpleasant or unwanted drug effects. One participant recalled an experience with ketamine in which a ‘k-hole’ was mitigated through sex:

I wasn't having a great time with it [ketamine]. I found it very intense, a bit too much, and we actually…she actually suggested that we had sex to… almost like to kind of ground me. I think I was fucking sitting there in the bedroom watching YouTube videos. I think it was the video, Duran Duran's ‘Wild Boys’, I had on repeat for some reason...Yes...we had quite gentle sex that…yes it sort of let my mind focus on the [thing], which I've heard people do with acid...Like if when you're, “Whoa,” and your mind starts flying off all over the place, it's almost like having that quite familiar sexual contact, you can always focus on that and it can sort of pull your brain back in.

(Cyril, M41, Bisexual/Pansexual, London - Study 1)

Another participant noted this effect in the context of using psychedelics:

That they, partly also I guess from my experience of having taken psychedelics anyway...the fact that they increase the intimacy between people and I'd also read actually that actually having sex is a good way of helping to control the trip if it's becoming I guess too challenging, and it's a useful way of grounding yourselves and making it more positive.

(Harry, M30, Heterosexual, London - Study 1)

As the quotations above demonstrate, engaging in sex might itself be conceived as a form of ‘peer-care’ (Pienaar et al., 2020a) around drug use that could mitigate the harms of a bad experience. While a minority of studies of chemsex have noted its potential for facilitating collective intimacy (Hakim, 2019), the rituals surrounding drug use are seldom incorporated in studies of enhancement (for an exception, see Vittelone, 2003). As our research suggests, we should not discount the possibility that practices of risk-management, peer-care and harm reduction around drug use can themselves be important facets of an enhanced sexual experience (Race, 2008).

## Conclusion

In this article, we have demonstrated that focus on narratives of risk and harm does not capture the full story of participants’ lived experience of sex-related drug use, but to focus solely on pleasure is also insufficient. By expanding our focus beyond the sex-related drug use of specific social groups, our analysis has revealed what individuals *across* genders and sexualities might gain from chemically enhanced sexual experiences, and that this cannot be reduced to an either/or of reparation of sexual problems or hedonism. We have provided evidence to suggest that sex-related drug use is not confined to various ‘scenes’ or sexual subcultures, and instead shown that the intentional use of drugs with sex also permeates normalised (Parker et al., 1998) or ‘recreational’ patterns of drug use beyond LGBTQ populations. While existing research has tended to focus on drugs that have been culturally established as ‘sexual enhancers’, such as GHB/GBL, mephedrone, methamphetamine and cocaine, our data corroborates recent research indicating that a much wider range of substances including MDMA/Ecstasy, LSD, and cannabis are also purposefully selected for experimentation in sexual contexts (Pienaar, Murphy, Race, & Lea, 2020a).

Through attending to enhancement, we have highlighted how biomedical conceptions of enhanced sex, whether in reference to physical function or libido, provide insufficient insight into pharmacosexual experimentation. Participants’ talk, while often disclosing cognition of pharmaceutical modification of drive, desire and function, frequently referred to forms of enhancement that could not be reduced to measurable effects. As Preciado (2008) has noted, pharmaceutical markets adapt rapidly to cater to new and shifting desires for sexual enhancement. In this context, the administration of drugs as a process of sexual curation reopens debates about the extent to which neoliberal narratives of self-improvement informed by these markets ‘inhabit’ sexuality in the twenty-first century. It is perhaps no coincidence that the narrative of achievement of ‘better sex’ through augmentation with drugs was particularly prevalent amongst those who identified as women in our studies. Women were more likely to draw implicitly on therapeutic discourses to explain their sex-related drug use, often as a means of overcoming inhibitions or to counter difficulties they had enjoying sex. They were also more likely to discuss regret and shame, sometimes expressing a dissonance between sexual activity they engaged in while on drugs, and who they ordinarily perceived their sexual ‘selves’ to be.

While narratives of self-actualisation were prevalent, as our analysis has revealed, pharmacosex also has the potential to open up an alternative horizon in which ‘the various mechanisms of sexuality’ are rejected and enable practices which instead ‘counter the grip of power with the claims of bodies, pleasures and knowledges, in their multiplicity and their possibilities of resistance’ (Foucault, 1978, p. 157). We might see here an important distinction between an additive and programmatic capitalist-biopolitical logic of sexual enhancement through drugs, and a more rhizomatic and radical logic of sex-drugs-bodies-pleasures in all their 'multiplicity'. Many of our interview subjects viewed sex-related drug use as a process of ‘struggling to achieve self-determination as techno-living bodies capable of joy and pleasure’ (Preciado, 2008, p. 304).

From a policy perspective, ignoring the purposeful ways in which broader populations enhance or alter their sexual practices in relation to drugs also risks an overemphasis on risk and harm (Pienaar et al., 2020a), which in turn perpetuates stigma towards individuals who engage in sex-related drug use. This is a particular concern for already marginalised groups, such as MSM engaging in chemsex, who are more likely to be subject to surveillance, over policing and criminalisation. Importantly, the current prioritisation of risk also minimises opportunities to communicate effective harm reduction messages to those who stand to benefit, and diminishes the potential of foregrounding ‘an appreciation of care as imminent in pleasure’ (Race, 2008, p. 422). Data portrays clear reflexivity among our participants, many of whom expressed frustrations at the preoccupation of public health campaigns focussed upon a specific population and mode of sex-related drug use. While our research has provided further evidence of the ‘persistence of pleasure’ (Measham, 2004), it has also exposed the limits of a mechanistic and ahistorical understanding of sexual enhancement as concerned purely with improved physical sexual performance and stamina, or augmentation of desire. Participants’ talk sometimes reflected these same binaries, but as our analysis has shown, a purely hedonic conceptualisation of pharmacosexual enhancement cannot be neatly distinguished from reparation and therapeutic use.

There is a dearth of harm reduction information that relates to how drug users might navigate some of the challenges related with using drugs beyond those traditionally associated with chemsex in sexual encounters. Moreover, that there exists a population of drug users who utilise recreational substances for purposes beyond hedonic consumption, and for their therapeutic benefits, currently appears to be overlooked by public health institutions. The diverse experiences and needs of these groups must be acknowledged if we are to provide effective sex education/sexual health information that is reflective of individuals’ lived experience. Our data provides strong evidence to suggest that there exists both the target population and the desire for these messages.

## Funding

Study 1 was supported by the 10.13039/100010269Wellcome Trust [Grant no: 207938/Z/17/Z].

## Declarations of Interests

None.

## References

[bib0002] ACMD (2007) GBL & 1,4-BD: Assessment of Risk to the Individual and Communities in the UK. Available at: https://assets.publishing.service.gov.uk/gover-nment/uploads/system/uploads/attachment_data/file/119047/report-on-gbl1.pdf.

[bib104] Aldridge A (2020). Intoxicating the “charmed circle”: Constructions of deviance and normativity by people who combine drugs and sex.. Criminology and Criminal Justice.

[bib0003] Anderson K., Reavey P., Boden Z. (2019). ‘Never drop without your significant other, cause that way lies ruin’: The boundary work of couples who use MDMA together. International Journal of Drug Policy.

[bib0004] Barad K. (2007). Meeting the universe halfway: Quantum physics and the entanglement of matter and meaning.

[bib0005] Barker M.J., Gill R., Harvey L. (2018). Mediated intimacy: Sex advice in media culture.

[bib0006] Barratt M.J., Maddox A. (2016). Active engagement with stigmatised communities through digitral ethnography.

[bib0007] Barratt M.J., Ferris J.A., Lenton S. (2015). Hidden populations, online purposive sampling, and external validity: Taking off the blindfold. Field Methods.

[bib0008] Bauman Z (2003). Liquid love: On the frailty of human bonds.

[bib0009] Beck J., Rosenbaum M. (1994). Pursuit of ecstasy: The MDMA experience. SUNY Press.

[bib92] Becker H (1953). Becoming a marihuana user.. American journal of Sociology,.

[bib0010] Bellis M.A., Hughes K., Calafat A., Juan M., Ramon A., Rodriguez J.A., Mendes F., Schnitzer S., Phillips-Howard P. (2008). Sexual uses of alcohol and drugs and the associated health risks: a cross sectional study of young people in nine European cities. BMC Public Health.

[bib0011] Bishop S. (2018). Chemsex and PrEP reliance are fuelling a rise in syphillis amongst men who have sex with men. The Conversation.

[bib99] Bøhling F (2017). Psychedelic pleasures: An affective understanding of the joys of tripping. International Journal of Drug Policy.

[bib0012] Bostrom N., Sandberg A. (2009). Cognitive enhancement: Methods, ethics, regulatory challenges. Science and Engineering Ethics.

[bib0013] Bourne A., Reid D., Hickson F., Torres Rueda S., Weatherburn P. (2014). The chemsex study: Drug use in sexual among gay and bisexual men in Lambeth, Southwark & Lewisham.

[bib0014] Boys A., Marsden J., Strang J. (2001). Understanding reasons for drug use amongst young people: a functional perspective. Health Education Research.

[bib0015] Braun V., Clarke V., Cooper H., Camic P.M., Long D.L., Panter A.T., Rindskopf D., Sher K.J. (2012).

[bib98] Cavanagh J, Smith T, Nordstrom K, Wilson M (2018). GHB, GBL Intoxication. Quick Guide to Psychiatric Emergencies.

[bib0016] Conrad P., Potter D. (2004). Human growth hormone and the temptations of biomedical enhancement. Sociology of Health and Illness.

[bib0017] Dean B.V., Stellpflug S.J., Burnett A.M., Engebretsen K.M. (2013). 2C or not 2C: Phenethylamine designer drugs review. Journal of Medical Toxicology.

[bib0018] Decary-Hetu D., Aldridge J. (2015). Sifting through the net: Monitoring of online offenders by researchers. European Review of Organised Crime.

[bib97] Dennis F (2019). *Injecting Bodies in More-than-Human Worlds: Mediating Drug-Body-World Relations*.

[bib0020] Dispatches. (2019). Sex, Drugs and Murder. Channel 4 Television. Broadcast date: 28th September.

[bib96] Dilkes-Frayne E (2014). Tracing the “event” of drug use: “Context” and the coproduction of a night out on MDMA. Contemporary Drug Problems.

[bib0021] Dotheé N. (2020). ‘Gay ‘chemsex’ culture in Hollywood almost killed me: this is how I survived’. NBC News.

[bib0024] Dyck E. (2008). Psychedelic psychiatry: LSD from clinic to campus.

[bib0025] Dymock A., Mechen B., Moyle L. (2019). Acid and the sexual psychonauts. Wellcome Collection.

[bib0027] Fisher M.L., Worth K., Garcia J.R., Meredith T. (2012). Feelings of regret following uncommitted sexual encounters in Canadian university students. Culture, Health & Sexuality.

[bib102] Foucault M (1978). The history of sexuality: Vol. 1. An introduction (R. Hurley, Trans)..

[bib0028] Giddens A. (1992). The transformation of intimacy: Sexuality, love and eroticism in modern societies.

[bib0029] Gill R. (2007). Postfeminist media culture: Elements of a sensibility. European Journal of Cultural Studies.

[bib0031] Hakim J. (2019). The rise of chemsex: Queering collective intimacy in neoliberal London. Cultural Studies.

[bib0032] Halberstam J. (1998). Female masculinity.

[bib0034] Hegazi A., Lee M.J., Whittaker W., Green S., Simms R., Cutt R., Nagington M., Nathan B., Pakianathan M.R. (2017). Chemsex and the city: sexualised substance use in gay bisexual and other men who have sex with men attending sexual health clinics. International Journal of STD and AIDS.

[bib0035] Hibbert MP., Brett CE., Porcellato LA., Hope VD. (2019). Psychosocial and sexual characteristics associated with sexualised drug use and chemsex among men who have sex with men (MSM) in the UK. Sexually Transmitted Infections.

[bib0036] Hodgson N. (2019). ‘So Brits have a lot of sex on drugs – why?. The Guardian.

[bib0037] HM Government. (2017). 2017 Drug Strategy. Retrieved 15th February 2020 from https://assets.publishing.service.gov.uk/government/uploads/system/uploads/att-achment_data/file/628148/Drug_strategy_2017.PDF.

[bib0038] Jacobs J. (2019). 'They broke my mental shackles': could magic mushrooms be the answer to depression. The Guardian.

[bib0039] Jozkowski K.N., Wiersma J.D. (2015). Does drinking alcohol prior to sexual activity influence college students’ consent?. International Journal of Sexual Health.

[bib0041] Kapitány-Fövény M., Mervo B., Corazza O., Kokonyei G., Farkas J., Urbán R., Zacher G., Demetrovics Z. (2015). Enhancing sexual desire and experience: An investigation of the sexual correlates of gamma-hydroxybutyrate (GHB) use. Human Psychopharmacology.

[bib0043] Kneale D., French R., Spandler H., Young I., Purcell C., Boden Z., Brown S., Callwood D., Carr S., Dymock A., Eastham R., Gabb J., Henley J., Jones C., McDermott E., Mkhwanazi N., Ravenhill J., Reavey P., Scott R., Smith C., Smith M., Tingay K., Thomas J. (2019). Conducting sexualities research: An outline of emergent issues and case studies from ten Wellcome-funded projects. Wellcome Open Research.

[bib0044] Lawn W., Aldridge A., Xia R., Winstock A.R. (2019). Substance-Linked Sex in Heterosexual, Homosexual, and Bisexual Men and Women: An Online, Cross-Sectional “Global Drug Survey” Report. The Journal of Sexual Medicine.

[bib0045] MAPS (2002). Talking with Ann and Sasha Shulgin. On the Existence of God and the Pleasures of Sex Drugs. Sex, Spirits and Psychedelics. MAPS.

[bib0046] McRobbie A. (2007). Top Girls? Young women and the postfeminist sexual contract. Cultural Studies.

[bib0047] Measham F. (2004). The decline of ecstasy, the rise of ‘binge’ drinking and the persistence of pleasure.. Probation Journal.

[bib0048] Mechen B. (2020). Sex, drugs and pharmacy. Pharmacy in History.

[bib0049] MENRUS (2018). About Chemsex. MENRUS. Retrieved 27th February 2020 from https://menrus.co.uk/drugs/about-chemsex/.

[bib94] Ma R, Perera S (2016). Safer “chemsex”: GPs’ role in harm reduction for emerging forms of recreational drug use. British Journal of General Practice.

[bib0052] Moncrieff M. (2018). Towards a supportive policy and commissioning environment for chemsex in England. Sex Health.

[bib0055] Mugford S. (1998). Pathology, pleasure, profit and the state: Towards an integrated theory of drug use. Proceedings of the Australian and New Zealand Society of Criminology Annual Conference, Sydney, August 22.

[bib0056] Nutt D. (2019). Psychedelic drugs - A new era in psychiatry. Dialogues in Clinical Neuroscience.

[bib0058] Palamar J.J., Griffin-Tomas M., Acosta P., Ompad D.C., Cleland C.M. (2018). A comparison of self-reported sexual effects of alcohol, marijuana, and ecstasy in a sample of young adult nightlife attendees. Psychology & Sexuality.

[bib105] Parent N, Ferlatte O, Milloy M.J, Fast D, Knight R (2020). The sexualised use of cannabis among young sexual minority men: “I’m actually enjoying this for the first time”. Culture, Health & Sexuality.

[bib101] Parker H, Aldridge J, Measham F (1998). *llegal leisure: The normalization of adolescent recreational drug use*.

[bib0060] Peugh J., Belenko S. (2001). Alcohol, drugs and sexual function: A review. Journal of Psychoactive Drugs.

[bib0061] Pienaar K., Murphy D.A., Race K., Lea T. (2020). Drugs as technologies of the self: Enhancement and transformation in LGBTQ cultures. International Journal of Drug Policy.

[bib0062] Pienaar K., Murphy D., Race K., Lea T., Hutton F. (2020). Sexualities and Intoxication: “To be intoxicated is to still be me, just a little blurry” - Drugs, enhancement and transformation in lesbian, gay, bisexual, transgender and queer cultures. Cultures of intoxication.

[bib0063] Preciado P.B. (2008). Testo junkie: Sex, drugs and biopolitics in the pharmacopornographic era.

[bib0064] Public Health England (2015). Substance misuse services for men who have sex with men involved in chemsex. Retrieved 8th January 2020 from: https://assets.publishing.service.gov.uk/government/uploads/system/uploads/att-achment_data/file/669676/Substance_misuse_services_for_men_who_have_sex_with-_men_involved_in_chemsex.pdf.

[bib100] Puffal E (2018). Sexualized drug use (“chemsex”) and high‐risk sexual behaviours in HIV‐positive men who have sex with men. HIV medicine.

[bib0065] Race K. (2008). The use of pleasure in harm reduction: Perspectives from the history of sexuality. International Journal of Drug Policy.

[bib0066] Race K. (2009). Pleasure consuming medicine: The queer politics of drugs.

[bib0067] Race K., Whelehan Patricia, Bolin Anne (2015). Sex and drugs. The international encyclopedia of human sexuality.

[bib0068] Race K. (2018). The gay science: Intimate experiments with the problem of HIV.

[bib0070] Rawson A.R., Washton A., Domier C.P., Reiber C. (2002). Drug and sexual effects: Role of drug type and gender. Journal of Substance Abuse Treatment.

[bib0071] Riessman C. (2005). ‘Narrative Analysis’, Narrative, memory & everyday life.

[bib0073] Ristuccia A, LoSchiavo C., Halkitis PN., Kapadia F. (2018). Sexualised drug use among sexual minority young adults in the United States: The P18 cohort study. International Journal of Drug Policy.

[bib0074] Rose N. (1990). Governing the soul: The shaping of the private self.

[bib0075] Rose N. (2001). The politics of life itself. Theory, Culture and Society.

[bib103] Segady T (2014). The utility of Weber’s ideal type: Verstehen and the theory of critical mass. Sociological Spectrum.

[bib0076] Sessa B. (2005). Can psychedelics have a role in psychiatry again. British Journal of Psychiatry.

[bib0077] Sessa B. (2007). Is there a case for MDMA Psychotherapy in the UK. Journal of Psychopharmacology.

[bib0078] Stevens O., Moncrieff M., Gafos M. (2020). Chemsex-related drug use and its association with health outcomes in men who have sex with men: a cross-sectional analysis of Antidote clinic service data. Sexually Transmitted Infections.

[bib0079] Strudwick P. (2020). The UK is ramping up its efforts to tackle a wave of chemsex crimes. Buzzfeed.

[bib93] Sessa B (2012). *The psychedelic renaissance: Reassessing the role of psychedelic drugs in 21st century psychiatry and society*.

[bib0080] Southworth P (2020). Psychedelics should be used to treat alcoholism and depression, former chief drug adviser claims. The Telegraph.

[bib0081] Stuart D. (2016). A chemsex crucible: the context and the controversy. Journal of Family Planning and Reproductive Health Care.

[bib0082] Stuart, D., (2016b). Chemsex is a complicated public health issue. Indetectables. Retrieved 25th August 2020 from http://indetectables.es/david-stuart-chemsex-is-a-complicated-public-health-issue.

[bib0084] Sumnall H., Riley S., Conchon S., Beynon C., Cole J. (2007). An investigation of the subjective experiences of sex after drug or alcohol intoxication. Journal of Psychopharmacology.

[bib0085] Tiefer L. (2008). Prognosis: More Pharmasex. Sexualities.

[bib0086] Torjesen I. (2014). Ketamine helps a third of patients with treatment-resistant depression, finds small UK study. British Medical Journal.

[bib0087] Van de Ven K., Mulrooney K.J.D., McVeigh J., Van de Ven K., Mulrooney K.J.D., McVeigh J. (2020). An introduction to human enhancement drugs. Human enhancement drugs.

[bib0088] Vittelone N. (2003). The syringe as prosthetic. Body & Society.

[bib0089] Weatherburn P., Hickson F., Reid D., Torres-Rueda S., Bourne A. (2017). Motivations and values associated with combining sex and illicit drugs (‘chemsex’) among gay men in South London: findings from a qualitative study. Sexually Transmitted Infections.

[bib0090] Zemishlany Z., Aizenberg D., Weizman A. (2001). Subjective effects of MDMA ("Ecstasy") on human sexual function. European Psychiatry.

[bib95] Zinberg N (1984). Drug, Set, and Setting The Basis for Controlled Intoxicant Use.

